# Comparative Characterisation and In Vitro Metabolic Bioactivities of Fucoidan-Rich Extracts from Three South African Brown Macroalgae

**DOI:** 10.3390/molecules31162823

**Published:** 2026-08-13

**Authors:** Coleen E. Grobler, Blessing Mabate, Justin B. Safari, Brett I. Pletschke

**Affiliations:** Enzyme Science Programme (ESP), Department of Biochemistry, Microbiology and Bioinformatics, Rhodes University, Makhanda 6139, South Africa; coleen1521@gmail.com (C.E.G.); bmabate@gmail.com (B.M.); safaribazibuhejustin@gmail.com (J.B.S.)

**Keywords:** fucoidan, *Ecklonia species*, *Sargassum species*, α-glucosidase, pancreatic lipase, antioxidant

## Abstract

Fucoidans are sulphated polysaccharides from brown macroalgae that have attracted considerable interest due to their diverse biological activities and potential applications in metabolic health. In this study, fucoidan-rich extracts were obtained from three South African brown macroalgal species, *Ecklonia maxima*, *Ecklonia radiata*, and *Sargassum incisifolium*, and comparatively characterised using biochemical and physicochemical analyses. The extracts were evaluated for carbohydrate, sulphate, L-fucose, protein, polyphenol, and uronic acid contents, while molecular weight distribution, Fourier-transform infrared spectroscopy (FTIR), thermogravimetric analysis (TGA), and proton Nuclear Magnetic Resonance (^1^H NMR) spectroscopy were used to investigate structural characteristics. All extracts displayed features characteristic of fucoidans, including sulphated fucose-containing polysaccharides. Notable species-dependent differences were observed, with *S. incisifolium* exhibiting the highest sulphate (~24%) and polyphenol (~6.76%) contents, stronger sulphate-associated FTIR signals, greater molecular weight heterogeneity, and enhanced thermal stability. The fucoidan-rich extracts exhibited concentration-dependent pancreatic lipase and α-glucosidase inhibitory activities, together with DPPH radical-scavenging activity. At 1.0 mg/mL, *S. incisifolium* demonstrated approximately 66% DPPH radical scavenging and 49% pancreatic lipase inhibition. *S. incisifolium* and *E. radiata* exhibited strong α-glucosidase inhibition, with IC_50_ values of ~29 and ~46 µg/mL, respectively, compared to ~354 µg/mL for acarbose. The findings highlight South African brown macroalgae as promising sources of fucoidans and contribute to understanding the relationships among fucoidan composition, structure, and bioactivity.

## 1. Introduction

Marine ecosystems represent a rich and underexplored source of structurally diverse bioactive compounds with significant pharmaceutical, nutraceutical, and biomedical potential [1,2]. Among marine organisms, macroalgae have attracted increasing scientific and industrial attention due to their abundance of bioactive metabolites, including pigments, polyphenols, proteins, minerals, and complex polysaccharides [3,4]. Based on pigmentation and biochemical composition, macroalgae are classified into three major groups: brown (Phaeophyceae), red (Rhodophyta), and green algae (Chlorophyta) [5]. Brown macroalgae are particularly valuable because they are rich in polysaccharides such as alginate, laminarin, and fucoidan. These compounds exhibit antioxidant, anti-inflammatory, and antidiabetic activities, along with useful physicochemical properties such as water retention, viscosity, and gel formation, thereby supporting their applications in the food, pharmaceutical, cosmetic, and biomedical industries [4].

Fucoidan is a structurally complex sulphated heteropolysaccharide predominantly found in the cell walls of brown algae. These polysaccharides are primarily composed of sulphated L-fucose residues, although varying amounts of galactose, mannose, glucose, xylose, uronic acids, and acetyl groups may also be present depending on the algal species, harvesting conditions, and extraction methods employed [3,6]. Fucoidans exhibit substantial structural heterogeneity, with molecular weights typically ranging from approximately 10 to 1600 kDa [3,7]. Importantly, their physicochemical properties and associated biological activities are strongly influenced by extraction methodology, structural parameters such as molecular weight, monosaccharide composition, sulphate content, sulphation pattern, and branching degree [3,8]. Consequently, comprehensive physicochemical characterisation of fucoidan is essential for understanding and interpreting its bioactive potential.

Increasing evidence suggests that fucoidans possess a broad spectrum of biological activities, including antioxidant, anti-inflammatory, antimicrobial, anti-obesity, antidiabetic, anticoagulant, and anticancer effects [7,9]. Seaweed-derived polysaccharides, including fucoidans, are also being investigated as cosmetic and dermo-cosmetic ingredients for their antioxidant, anti-inflammatory, photoprotective, moisturising, and anti-ageing properties [10,11]. Several studies have highlighted that these bioactivities are closely linked to the structural characteristics of fucoidan, particularly molecular size and degree of sulphation [6,12]. Fucoidan composition and physicochemical profiles vary among algal species, while extraction conditions may preferentially recover fractions that differ in molecular weight, sulphation, and associated constituents. This variability complicates comparisons of biological activity and the establishment of clear structure–function relationships. FTIR, thermal analysis, molecular-weight determination and monosaccharide profiling provide complementary compositional and physicochemical information but are insufficient for complete structural elucidation, which requires additional techniques such as multidimensional NMR, GC–MS, and glycosidic-linkage analysis. Among the reported bioactivities of fucoidan, its potential role in the management of metabolic disorders has attracted considerable attention. Obesity and type 2 diabetes mellitus are closely associated with oxidative stress and dysregulated lipid and glucose metabolism [13]. Enzymes such as pancreatic lipase and α-glucosidase are important therapeutic targets involved in the digestion of dietary fat and carbohydrates, respectively [6,14].

South African brown macroalgae represent a potentially valuable yet comparatively underexplored source of fucoidan. *Ecklonia maxima*, *Ecklonia radiata*, and Sargassum species are abundant along the South African coastline and may yield fucoidans with unique structural and biological properties, driven by differences in environmental conditions, geographical distribution, and species-specific biosynthetic pathways. While some studies have investigated the biological properties of fucoidan from selected South African kelp species [8,15], further investigation of South African brown macroalgae remains warranted to explore species-specific structural variations and their associated bioactivities. Among the species selected, *E. maxima* is a large, surface-canopy-forming kelp that dominates rocky reefs along South Africa’s cool-temperate west coast and extends eastwards along the south coast [16]. Fucoidan extract preparations from *E. maxima* have demonstrated in vitro α-glucosidase inhibitory activity and effects on cancer cell adhesion, migration, and survival [7,8]. *E. radiata* is a smaller kelp, typically reaching approximately 2 m, and is an important habitat-forming species on warm-temperate reefs of the Southern Hemisphere, including South Africa [17]. Its fucoidan has been chemically characterised and shown to inhibit α-glucosidase, with enhanced inhibition observed when combined with acarbose [15]. In contrast, *S. incisifolium* is a branching non-kelp brown alga characterised by leaf-like blades, vesicles, and reproductive receptacles and occurs along South African rocky coasts [18]. Species-specific pharmacological evidence remains limited; however, polyphenol-containing aqueous extracts of *S. incisifolium* have demonstrated in vitro DPPH radical-scavenging activity [19]. These ecological, morphological, and reported biological differences provide a rationale for comparing the composition and biological activities of their fucoidan-rich extracts.

Therefore, this study aimed to characterise, in a comparative manner, the fucoidan-rich extracts obtained from three South African brown macroalgal species: *E. maxima*, *E. radiata*, and *S. incisifolium*. The fucoidan-rich extracts were subjected to chemical and physicochemical characterisation, including compositional analysis, molecular weight determination, FTIR, and 1H NMR, and their bioactive potential was evaluated through inhibition of pancreatic lipase and α-glucosidase and assessment of antioxidant activity. This study further aimed to explore potential relationships between the physicochemical characteristics of fucoidan-rich extracts and their associated biological activities, thereby contributing to the growing understanding of fucoidan structure–activity relationships and their potential applications as functional natural products for metabolic health.

## 2. Results and Discussion

### 2.1. Fucoidan-Rich Extracts Yield

Hot-water extraction recovered fucoidan-rich material from all three macroalgal species, with yields ranging from 3.73% to 8.76% relative to the dry weight of the depigmented seaweed. Extraction yields differed significantly among species (one-way ANOVA). *E. maxima* produced the highest yield of 8.76 ± 0.81%, which was approximately 2-fold higher than the yields obtained from *S. incisifolium* (3.92 ± 0.49%) and *E. radiata* (3.73 ± 0.54%), respectively. Tukey’s multiple-comparisons test confirmed that the *E. maxima* yield was significantly higher than that of both *S. incisifolium* and *E. radiata*. In contrast, the yields from *S. incisifolium* and *E. radiata* were not significantly different (*p* < 0.05). Using a comparable hot-water extraction method, Mabate et al. [7] previously reported yields of 5.4% and 5.9% for *E. maxima* and *E. radiata*. Therefore, the present *E. maxima* yield was higher, whereas the *E. radiata* yield was lower than those previously reported for these species. Fucoidan recovery can vary by species, geographic location, maturity, harvest season, and extraction conditions. A previous study demonstrated substantial variation in fucoidan content among different harvesting periods [20]. Nevertheless, because these variables were not systematically investigated in the present study, the observed differences cannot be attributed solely to harvesting time and likely reflect combined species-, environmental-, and batch-related variation.

The amounts extracted are in the range reported in the literature [7,10]. It should also be noted that the reported values represent the gravimetric yields of the recovered fucoidan-rich extracts. Extraction yields were therefore interpreted alongside subsequent compositional analyses of carbohydrates, L-fucose, sulphate, protein, polyphenols, uronic acids, and ash to assess the chemical characteristics of the recovered material.

### 2.2. Molecular Weight Distribution of Fucoidan-Rich Extracts

**Figure 1:**
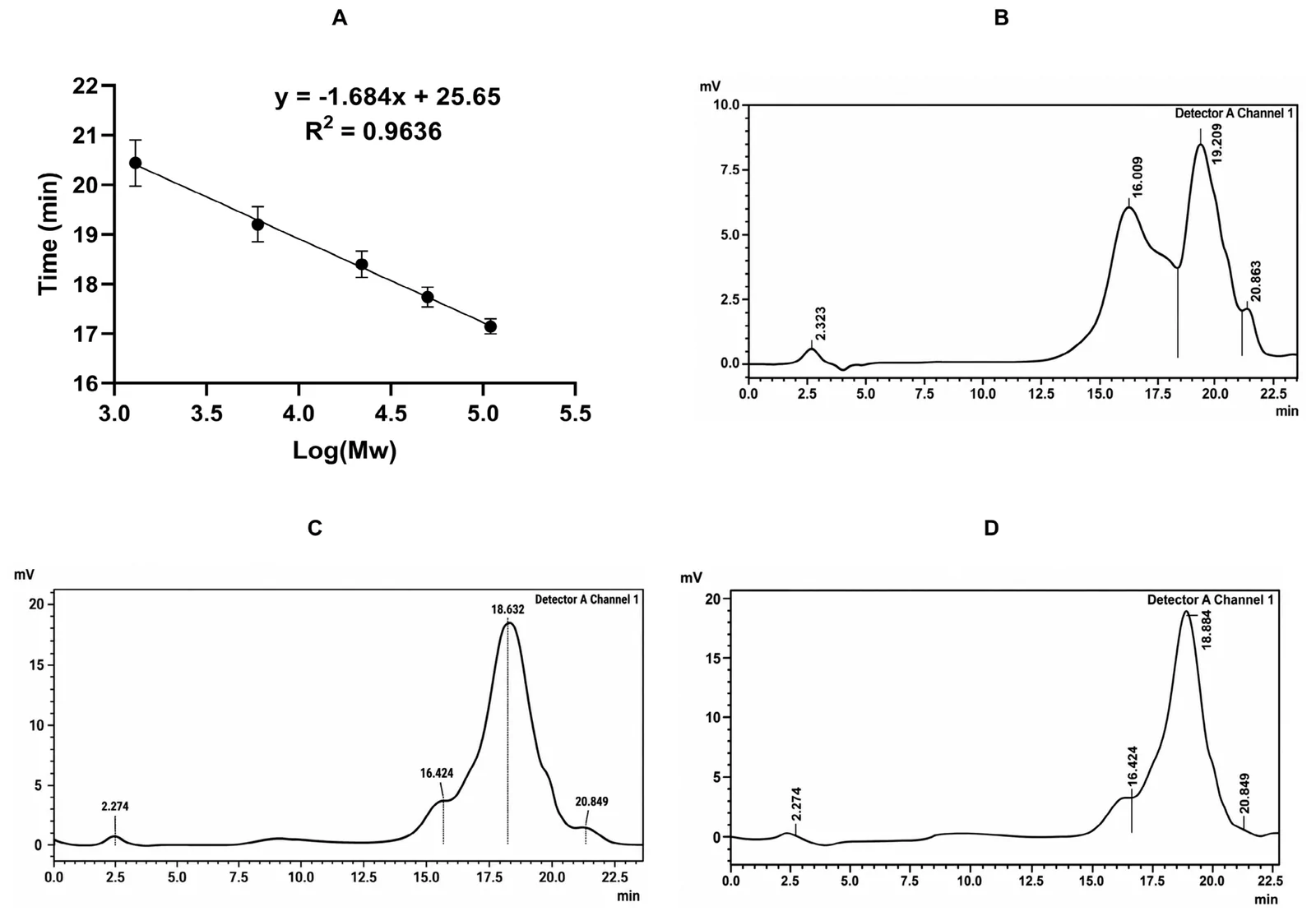
Molecular weight determination of fucoidan-rich extracts by size-exclusion HPLC-RID. (**A**) Calibration curve generated using pullulan standards. Chromatographic profiles of fucoidan-rich extracts from (**B**) *Sargassum incisifolium*, (**C**) *Ecklonia maxima*, and (**D**) *Ecklonia radiata*.

The molecular weight distributions of fucoidan-rich extracts from *S. incisifolium*, *E. maxima*, and *E. radiata* were determined by size-exclusion high-performance liquid chromatography coupled with refractive index detection (SEC-HPLC-RID). A calibration curve generated using pullulan standards demonstrated a linear relationship between retention time and molecular weight and was subsequently used to estimate the average molecular weights of the fucoidan-rich extracts (Figure 1). The chromatographic profile of *S. incisifolium* indicated a highly polydisperse molecular weight distribution comprising a major high-molecular-weight fraction ~450 kDa and a substantial lower-molecular-weight fraction ~5 kDa. The weighted-average molecular weight of the peak area was calculated to be 226 kDa. In contrast, the Ecklonia fucoidan-rich extracts exhibited a single major peak, with molecular weights of ~19 kDa and ~11 kDa for *E. maxima* and *E. radiata*, respectively. A previous study on *E. maxima* and *E. radiata* reported the fucoidans molecular weights (Mws) as ~27 kDa and ~8.5 kDa, respectively [7]. The Mws are close to each other, although the seaweed batches and ages differed. The Mws fall within the range reported for fucoidans from brown macroalgae, which have been shown to vary substantially by species, geographic origin, and extraction method [6,21,22]. MWs ranging from approximately 20 to 320 kDa have previously been reported for fucoidan fractions isolated from several brown macroalgal species [7,23].

Despite their similar average Mws, notable differences were observed in the chromatographic profiles of the extracts (Figure 1B–D). The fucoidan-rich extracts isolated from *E. maxima* and *E. radiata* displayed relatively narrow chromatographic peaks with similar elution patterns, suggesting comparatively homogeneous molecular weight distributions. In contrast, the *S. incisifolium* fucoidan-rich extract exhibited a broader chromatographic profile, comprising multiple peaks, including an earlier-eluting fraction corresponding to higher-molecular-weight material and a later-eluting fraction indicative of lower-molecular-weight components. This profile is characteristic of a polydisperse polysaccharide system containing multiple molecular populations.

Polydispersity is a common feature of fucoidans due to the structural complexity of these sulphated polysaccharides and their heterogeneous biosynthesis within brown algae [23,24]. Variations in chain length, branching patterns, degree of sulphation, and monosaccharide composition can result in multiple molecular weight populations within a single extract [25]. The broader molecular weight distribution observed for *S. incisifolium* therefore suggests a more heterogeneous fucoidan-rich structure than that of fucoidan extracted from the two *Ecklonia* species. Previous studies have demonstrated that molecular weight distribution may be as important as average molecular weight in determining fucoidan functionality. For example, Lahrsen et al. (2018) reported size-dependent biological activities among fucoidan fractions derived from *Fucus vesiculosus* [26], while Attjioui et al. (2021) demonstrated that fucoidan-rich brown algal extracts with differing molecular weight profiles exhibited distinct carbohydrate-digestive enzyme inhibitory activities [27]. Furthermore, molecular weight heterogeneity has been linked to variations in polysaccharide conformation, solution behaviour, and interactions with biological targets [23,25].

The higher average molecular weight and polydispersity of *S. incisifolium* fucoidan-rich suggest that the molecular weight distribution, rather than average molecular weight alone, may be the distinguishing feature from *Ecklonia* fucoidans. The presence of both higher- and lower-molecular-weight fractions in this extract may contribute to differences in physicochemical properties and biological activity compared with the more homogeneous fucoidans obtained from *E. maxima* and *E. radiata*. These findings further highlight the importance of evaluating fucoidan heterogeneity in addition to average molecular weight when investigating structure–function relationships.

### 2.3. Compositional Analysis of Fucoidan-Rich Extracts

#### 2.3.1. Chemical Characterisation

The chemical composition of fucoidan-rich extracts from *S. incisifolium*, *E. radiata*, and *E. maxima* is presented in Table 1. The chemical composition of the fucoidan-rich extracts differed among the investigated species. The apparent total carbohydrate content of the *S. incisifolium* extract was 38.50 ± 4.57 g GE/100 g dried extract, which was significantly lower than that of *E. radiata* (90.23 ± 3.69 g GE/100 g dried extract) and *E. maxima* (96.27 ± 4.79 g GE/100 g dried extract) (*p* < 0.05). The two *Ecklonia* extracts did not differ significantly. The high carbohydrate content observed for the two *Ecklonia* extracts are plausible for polysaccharide-enriched preparations and are consistent with our previous study, in which *E. maxima* and *E. radiata* fucoidan-rich extracts contained 72.8 ± 5.2% and 88.0 ± 7.4% total carbohydrate, respectively [7]. Nevertheless, the present values were determined colorimetrically using D-glucose as the calibration standard and are therefore expressed as glucose equivalents rather than direct gravimetric carbohydrate mass fractions. Differences in the colour responses of individual monosaccharides, together with potential matrix-related absorbance from co-extracted constituents, may affect the estimates. Because the chemical compositions of the extracts differ, these matrix effects cannot be assumed to be identical among the three species. The substantially higher carbohydrate content observed in *E. maxima* suggests a greater proportion of polysaccharide material within the extract. In contrast, the lower carbohydrate content of *S. incisifolium* may reflect the presence of additional sulphated and phenolic constituents.

The sulphate content varied considerably among species, ranging from 10.70% in *E. maxima* to 24.01% in *S. incisifolium* (Table 1). Sulphate groups are among the defining structural features of fucoidans and are largely responsible for many of their physicochemical and biological properties [24,28]. Sulphate contents within the range observed here have been reported previously for fucoidans isolated from brown seaweeds, although substantial interspecies variation is common [21,22].

L-fucose, the principal monosaccharide component of fucoidan, was detected in all extracts. Fucoidans from *E. radiata* (~55.86%) and *E. maxima* (~49.55%) contained substantially higher L-fucose levels than the *S. incisifolium* extract (~36.74%). High fucose contents are considered indicative of fucoidan-rich extracts and support the isolation of fucose-containing sulphated polysaccharides from all three species [24,29]. The combination of high fucose and sulphate contents observed in the *Ecklonia* extracts is characteristic of fucoidans reported from kelp species. In contrast, the comparatively lower fucose content and elevated sulphation observed in *S. incisifolium* may indicate structural differences in fucoidan composition between the genera *Sargassum* and *Ecklonia*. Only L-fucose was quantified in the present study. Therefore, the presence and relative abundance of other monosaccharides could not be established, and comprehensive monosaccharide profiling using HPAEC-PAD or GC–MS will be required for more detailed compositional characterisation.

Protein contents were low across all extracts, ranging from 4.76% to 6.75%. Protein is commonly regarded as a co-extracted contaminant during fucoidan isolation, and the relatively low protein levels observed indicate that the extraction and purification procedures effectively reduced protein impurities [22,29]. Similarly, uronic acid contents were low, ranging from 1.2% to 2.7%. Uronic acids are major constituents of alginates, which are among the principal contaminants encountered during fucoidan extraction from brown macroalgae [24]. The CaCl_2_ treatment, centrifugation, and mild ethanol precipitation successfully removed alginates. The low uronic acid levels, therefore, suggest limited alginate contamination and support the relative purity of the fucoidan-rich extract.

Polyphenol contents varied considerably between species, with *S. incisifolium* exhibiting the highest concentration (6.76%), followed by *E. radiata* (4.02%) and *E. maxima* (1.08%). Polyphenolic compounds, particularly phlorotannins, are frequently co-extracted from brown seaweeds and may contribute to the biological activities of fucoidan preparations through antioxidant mechanisms [28]. The elevated polyphenol content observed in *S. incisifolium* may therefore contribute to differences in bioactivity observed between the extracts. The compositional analysis confirmed the successful extraction of fucoidan-rich sulphated polysaccharides from all three brown macroalgal species. The extracts were characterised by high fucose and sulphate contents, together with relatively low protein and uronic acid levels, indicating limited contamination by non-fucoidan components. Notably, the *S. incisifolium* fucoidan differed from the *Ecklonia*-derived fucoidans through its higher sulphate and polyphenol contents, lower fucose content, and greater compositional heterogeneity, suggesting a structurally distinct fucoidan population.

#### 2.3.2. Structural Validation of Fucoidan-Rich Extracts

##### Fourier Transform Infrared Spectroscopy (FTIR) Analysis

**Figure 2:**
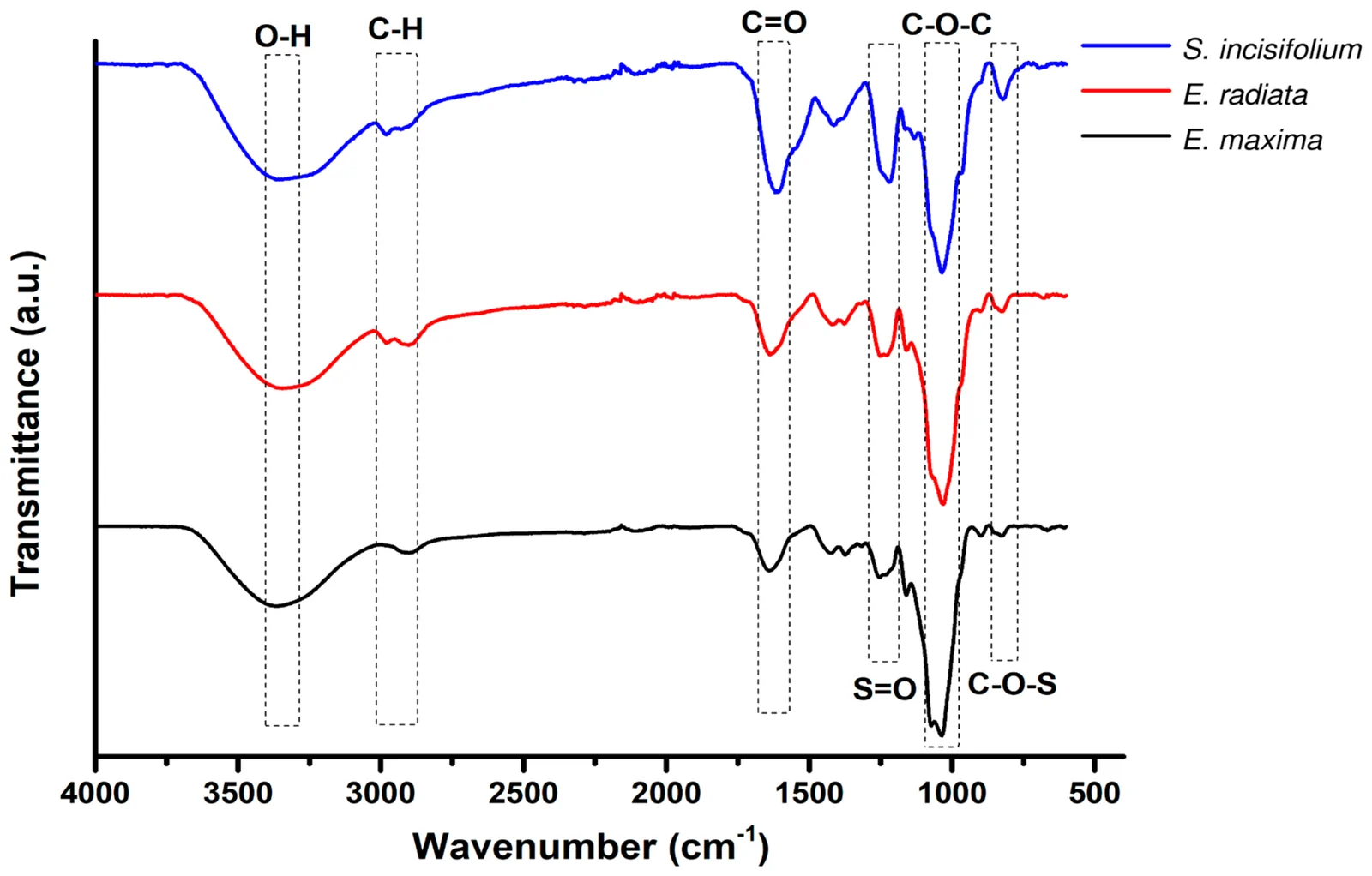
Fourier-transform infrared (FTIR) spectra of fucoidan-rich extract from *Sargassum incisifolium*, *Ecklonia radiata*, and *Ecklonia maxima* over the wavenumber range of 4000–600 cm^−1^.

The FTIR spectra of fucoidan-rich extracts from *E. maxima*, *E. radiata*, and *S. incisifolium* are presented in Figure 2. The spectra exhibited absorption bands characteristic of sulphated polysaccharides and were consistent with previously reported fucoidan profiles from brown macroalgae [7,30]. Broad absorption bands observed around 3359 cm^−1^ in all extracts were attributed to O–H stretching vibrations associated with hydroxyl groups within polysaccharide structures and hydrogen-bonded water molecules. Similar hydroxyl-associated bands have been widely reported in fucoidans isolated from brown seaweeds and are considered characteristic of carbohydrate-rich polymers [7].

Absorption bands observed within the 2900–3000 cm^−1^ region were assigned to C–H stretching vibrations associated with pyranose rings and methyl groups of carbohydrate entities, including fucose residues [31]. The bands observed around 1600–1700 cm^−1^ corresponded to carbonyl (C=O) stretching vibrations and O-acetyl group stretching, which are commonly associated with acetylated polysaccharide components present within fucoidan extracts [10]. Characteristic sulphate-associated absorption bands were detected on all the fucoidan-rich samples between 1210 and 1270 cm^−1^. These peaks are attributed to asymmetric S=O stretching vibrations and are considered a defining spectral feature of sulphated fucoidans [10]. Furthermore, absorption bands within the 1040–1020 cm^−1^ region, observed in all the fucoidan-rich samples Figure 2, corresponded to stretching vibrations of glycosidic C–O–C bonds within the polysaccharide backbone structure [7,10]. Peaks observed between 820 and 840 cm^−1^ were attributed to C–O–S bending vibrations associated with sulphate substitution on sugar residues, further confirming the sulphated nature of the extracted polysaccharides [10,30]. Although the fucoidan-rich extracts exhibited similar FTIR profiles, subtle differences in peak intensities and band distributions suggested structural variability among species. In particular, variations within the sulphate-associated spectral regions may indicate differences in the degree of sulphation and substitution patterns, which are known to influence fucoidan physicochemical properties and biological activities. The FTIR spectra showed absorption bands consistent with functional groups commonly associated with fucoidan-rich sulphated polysaccharides in extracts obtained from the investigated South African brown macroalgae.

##### Proton NMR Analyses of Fucoidan-Rich Extracts

**Figure 3:**
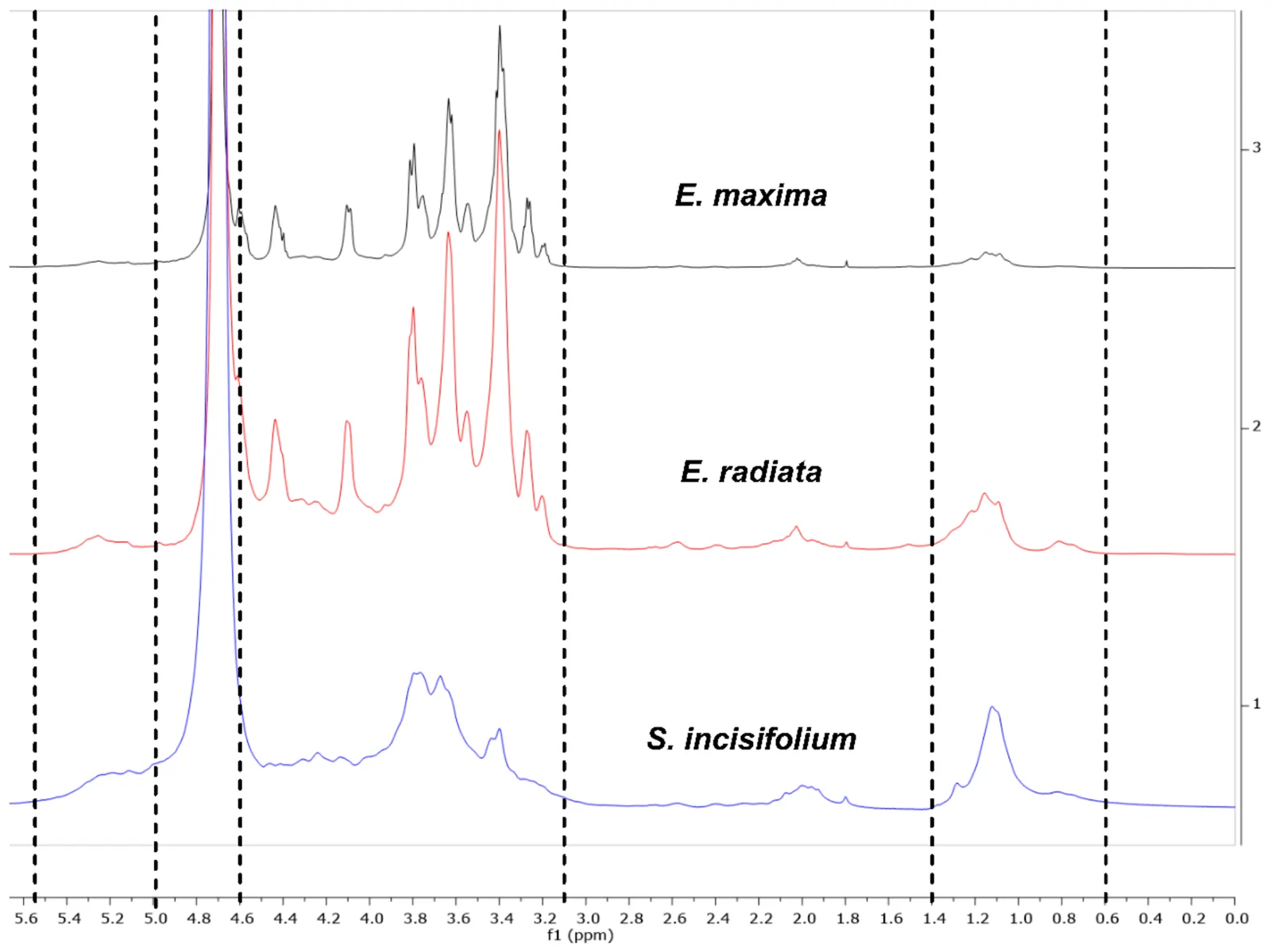
^1^H NMR spectra of fucoidan-rich extracts from *Sargassum incisifolium*, *Ecklonia maxima*, and *Ecklonia radiata*, showing characteristic proton resonances associated with fucose-containing sulphated polysaccharides (fucoidan).

The structural features of the fucoidan-rich extracts were further investigated by 1H NMR spectroscopy (Figure 3). All three extracts exhibited spectral characteristics typical of brown seaweed fucoidans, confirming the presence of sulphated fucose-containing polysaccharides. Similar proton NMR profiles have previously been reported for fucoidans isolated from *Ecklonia*, *Sargassum*, *Fucus*, and other brown macroalgae [7,32]. Signals observed between approximately 1.1 and 1.5 ppm correspond to the methyl protons (H-6) of α-*L*-fucopyranose residues, which are regarded as characteristic markers of fucoidan structures [32,33]. The presence of these peaks in all samples confirms that fucose is a major structural component of the extracted polysaccharides. Consistent with the FTIR spectra (Figure 2), which showed characteristic sulphate-associated absorptions at approximately 1250 and 840 cm^−1^, the methyl proton signals were most pronounced in *S. incisifolium*, supporting the biochemical analysis (Table 1) that demonstrated substantially higher sulphate levels (24.01%) than in *E. radiata* (15.27%) and *E. maxima* (10.70%).

The region between approximately 3.2 and 4.5 ppm contained multiple overlapping resonances broadly attributable to carbohydrate ring protons [32,33]. However, because only L-fucose was quantified and substantial signal overlap occurs in this region, the resonances could not be assigned confidently to specific additional monosaccharides. Although galactose, mannose and glucose have previously been reported in fucoidans from *Ecklonia* and related *Sargassum* species [7], their presence and relative abundance in the current extracts require confirmation through comprehensive monosaccharide profiling. The spectra of *E. maxima* and *E. radiata* displayed more numerous and better-resolved signals in this region than *S. incisifolium* (Figure 3), indicating a composition of carbohydrate residues. This observation agrees with the compositional analysis, which showed that *E. maxima* had the highest total carbohydrate content (~96%) and *E. radiata* had the highest fucose content (55.86%), whereas *S. incisifolium* exhibited substantially lower total carbohydrate levels (~38%).

Notably, the spectrum of *S. incisifolium* appeared broader and less resolved than that of *Ecklonia* fucoidan-rich extracts. This broadening likely reflects a more heterogeneous polysaccharide population and is consistent with the size-exclusion chromatographic analysis, which revealed a polydisperse molecular weight distribution. In contrast, *E. maxima* and *E. radiata* exhibited comparatively uniform chromatographic profiles, with a single dominant molecular-weight fucoidan (Figure 1; Table 1). The coexistence of both larger- and smaller-molecular-weight fractions within *S. incisifolium* suggests a more complex fucoidan architecture, potentially arising from variations in sulphation patterns, branching, or the incorporation of additional monosaccharide residues, characteristics commonly reported for *Sargassum*-derived fucoidans [34]. A weak signal around 2.0–2.2 ppm was also observed and may be attributed to acetylated sugar residues or methylated fucose units, features frequently reported in fucoidans from *Sargassum* species [32]. The greater prominence of this region in *S. incisifolium* may indicate a higher degree of structural substitution than in *Ecklonia* fucoidans.

Importantly, no prominent resonances were observed in the region typically associated with uronic acid-rich polysaccharides (approximately 5.5–6.0 ppm), consistent with the biochemical data, which showed low uronic acid content in all extracts (1.2–2.7%). Likewise, the absence of additional unexpected resonances suggests minimal contamination by other algal polysaccharides such as alginate, indicating that the extraction and purification procedures yielded relatively fucoidan-enriched preparations. The ^1^H NMR spectra confirmed that all extracts contained sulphated fucose-rich polysaccharides characteristic of fucoidan. However, clear structural differences were evident between genera. The *Ecklonia* fucoidans exhibited highly similar spectral profiles, reflecting comparable carbohydrate-rich compositions and molecular weight distributions. In contrast, *S. incisifolium* displayed a more heterogeneous spectrum characterised by stronger sulphate-associated signals, broader resonances, and increased structural complexity. These observations are consistent with the higher sulphate, elevated ash, and greater polyphenol content, and the polydisperse molecular weight profile of the *S. incisifolium* extract, collectively indicating a structurally distinct fucoidan-rich extract compared with the *Ecklonia*-derived fucoidans. Although inferences were made for the purpose of comparing our extracts, the overlapping 1D ^1^H NMR signals do not permit confident determination of glycosidic linkage, branching, or sulphation patterns. These features would require methylation–GC–MS and complementary two-dimensional NMR analysis.

##### Thermogravimetric Analysis of Fucoidan-Rich Extracts

**Figure 4:**
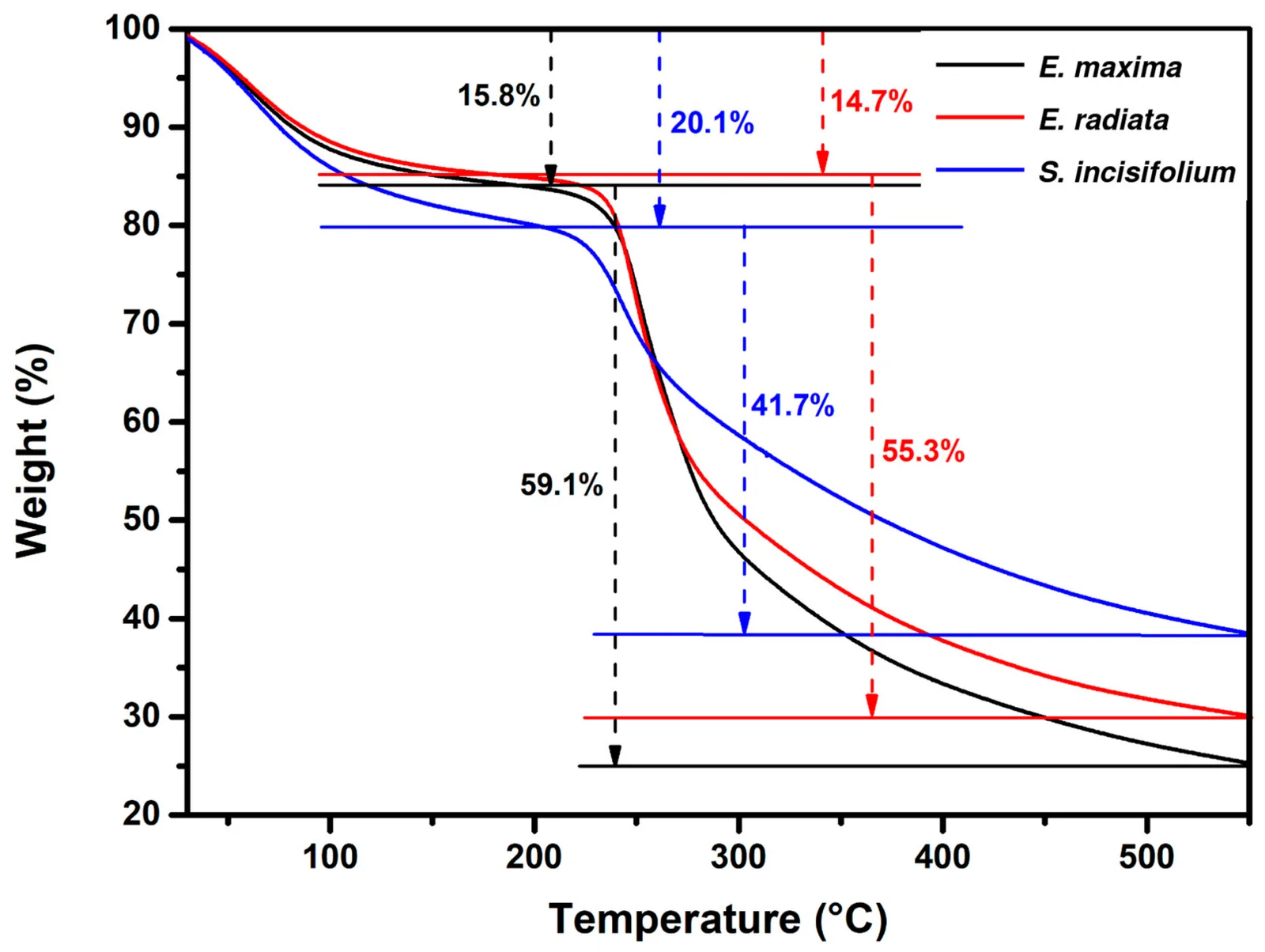
Thermogravimetric analysis (TGA) thermograms of fucoidans extracted from *Sargassum incisifolium*, *Ecklonia radiata*, and *Ecklonia maxima*. The thermograms illustrate the thermal stability and decomposition behaviour of the fucoidan-rich extracts over the temperature range of 30–550 °C under an inert nitrogen atmosphere.

Thermogravimetric analysis (TGA) was performed to evaluate the thermal stability and decomposition behaviour of the fucoidan-rich extract (Figure 4). The thermograms of fucoidan-rich extracts obtained from *S. incisifolium*, *E. radiata*, and *E. maxima* exhibited similar decomposition profiles characteristic of sulphated polysaccharides, confirming the polymeric nature of the extracted compounds [7,35]. All samples exhibited two major stages of thermal degradation. The initial weight-loss stage occurred between 30 and approximately 200 °C, accounting for 20.1%, 14.7%, and 15.8% mass loss in *S. incisifolium*, *E. radiata*, and *E. maxima*, respectively (Figure 4). This initial mass reduction is commonly attributed to the evaporation of physically adsorbed and bound water molecules, as well as the loss of low molecular weight volatile compounds associated with polysaccharide matrices [36]. The higher moisture-associated weight loss observed in *S. incisifolium* suggests a greater capacity for water retention, which may be related to differences in polysaccharide composition, sulphate substitution, or molecular architecture.

The second and most significant degradation stage occurred between approximately 205 °C and 550 °C and corresponded to the thermal decomposition and carbonisation of the polysaccharide backbone. During this phase, *S. incisifolium* exhibited the lowest loss in mass (41.7%), followed by *E. radiata* (55.3%) and *E. maxima* (59.1%). Similar degradation behaviour has been reported for fucoidans isolated from other brown macroalgae, where thermal decomposition within this temperature range is associated with depolymerisation reactions, cleavage of glycosidic linkages, and degradation of carbohydrate constituents [7,37]. The lower mass loss observed for *S. incisifolium* suggests a smaller proportion of thermally degradable organic material relative to the *Ecklonia*-derived fucoidans.

Although all extracts displayed broadly similar thermal degradation profiles, notable species-dependent differences were observed. *Sargassum incisifolium* exhibited the lowest mass loss (61.8%) and the highest residual mass (ash content) (38.2%) at 550 °C, compared with *E. radiata* (30.0%) and *E. maxima* (25.1%), indicating greater thermal stability of the *Sargassum*-derived fucoidan. This observation is consistent with the FTIR spectra, where *S. incisifolium* fucoidan-rich extract displayed more pronounced sulphate-associated absorption bands within the 1210–1270 cm^−1^ and 820–840 cm^−1^ regions, suggesting a higher degree of sulphation relative to the *Ecklonia* fucoidan-rich extracts.

In addition, size-exclusion chromatography analysis revealed a broader molecular weight distribution and evidence of polydispersity in *S. incisifolium* fucoidan-rich extract (Figure 1), whereas the fucoidans from *E. radiata* and *E. maxima* exhibited more homogeneous molecular weight profiles. Previous studies have shown that the thermal behaviour of fucoidan is influenced by multiple structural characteristics, including sulphation degree, molecular weight, and polymer organisation [7,24]. The enhanced thermal stability observed for *S. incisifolium* fucoidan-rich extract may therefore reflect the combined effects of increased sulphation (Table 1) and a more heterogeneous polysaccharide structure (Figure 1). Following decomposition of the organic fraction, the remaining mass was attributed to thermally stable inorganic constituents, including sulphates, phosphates, and mineral salts, collectively known as the ash content. Collectively, the FTIR, TGA, and molecular weight analyses suggest that the *S. incisifolium* fucoidan-rich extract possesses physicochemical characteristics distinct from those of the *Ecklonia*-derived fucoidans, which may contribute to the differences observed in their biological activities.

### 2.4. Biological Activity of Fucoidan-Rich Extracts

#### 2.4.1. Fucoidan-Rich Extracts Potential in Inhibiting Pancreatic Lipase

**Figure 5:**
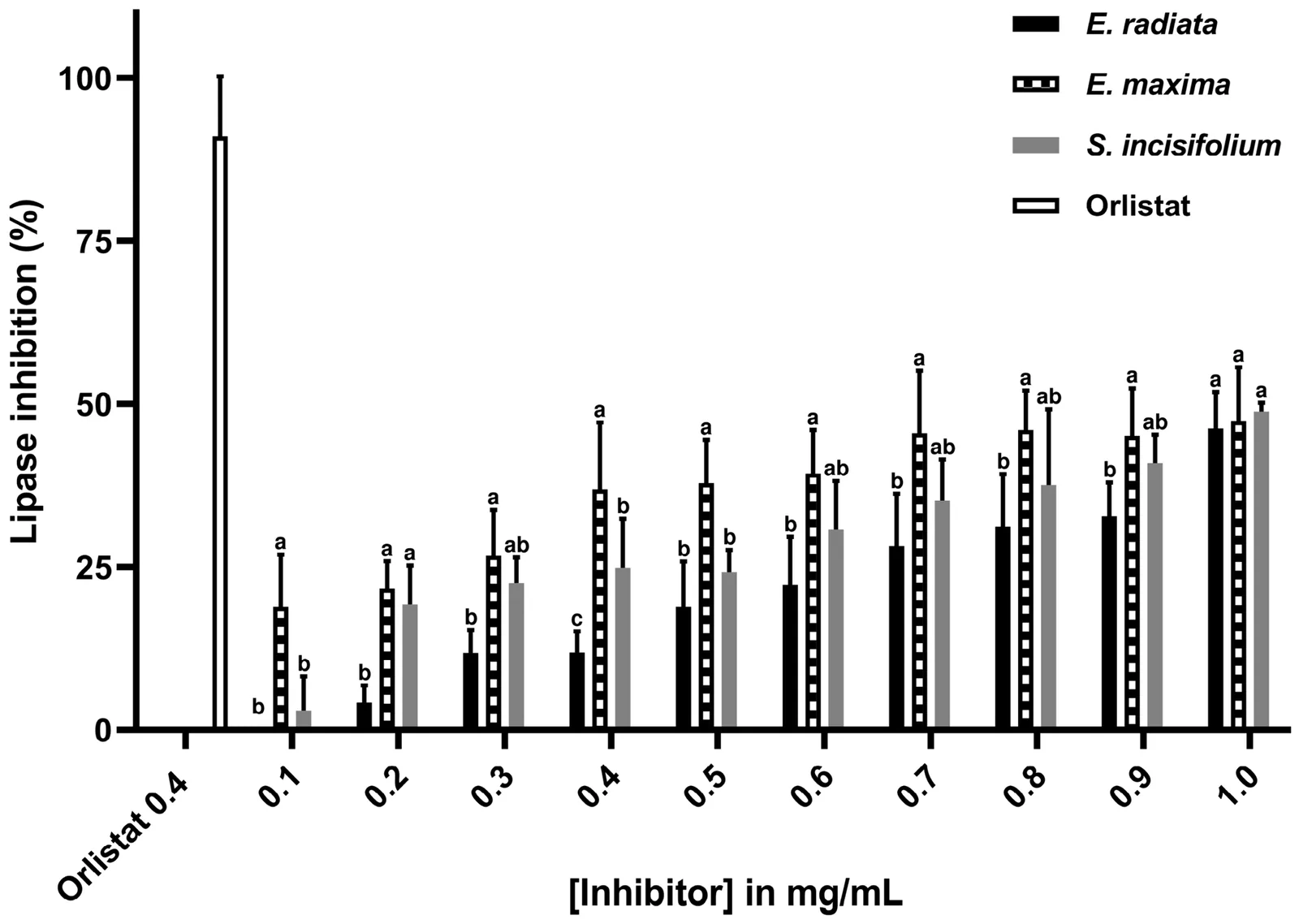
Dose-dependent inhibition of pancreatic lipase by fucoidan-rich extracts (0.1–1.0 mg/mL) from *Ecklonia radiata*, *Ecklonia maxima* and *Sargassum incisifolium*. Data are presented as mean ± SD (*n* = 4). Differences among extracts at each concentration were analysed using two-way ANOVA followed by Tukey’s multiple-comparisons test. Bars sharing the same letter are not significantly different, whereas different letters indicate significant differences (*p* < 0.05).

The inhibitory effects of fucoidans extracted from *E. maxima*, *E. radiata*, and *S. incisifolium* on pancreatic lipase activity are presented in Figure 5. All fucoidan-rich extracts exhibited dose-dependent inhibition of pancreatic lipase, with significantly lower enzyme activity observed at increasing fucoidan concentrations compared to the uninhibited control (*p* < 0.05). At the highest tested concentration (1.0 mg/mL), the residual pancreatic lipase activities were ~52.5%, ~53.7%, and ~51.2% for *E. maxima*, *E. radiata*, and *S. incisifolium*, respectively, corresponding to approximately 47–49% inhibition. While *E. radiata* and *S. incisifolium* exhibited inhibitory activities approaching that of orlistat only at concentrations above 0.8 mg/mL, *E. maxima* demonstrated comparable inhibition from 0.4 mg/mL, indicating greater activity at lower concentrations.

Pancreatic lipase is the principal enzyme responsible for the hydrolysis of dietary triglycerides into absorbable fatty acids and monoglycerides. Consequently, inhibition of this enzyme is a well-established strategy for reducing dietary fat absorption and managing obesity [38]. The significant dose-dependent inhibition observed across all three fucoidan-rich extracts, therefore, suggests their potential as natural modulators of lipid digestion. The inhibitory activities observed in the present study are consistent with previous reports describing pancreatic lipase inhibition by fucoidans isolated from brown macroalgae. Lu et al. (2024) reported dose-dependent pancreatic lipase inhibition by fucoidan extracted from *Laminaria japonica*, while Zhang et al. (2024) demonstrated that purified fucoidan fractions from the same species inhibited pancreatic lipase by approximately 42–49% at concentrations comparable to those used in this study [39,40]. Similarly, Magwaza and Islam (2023) highlighted pancreatic lipase inhibition as one of the principal mechanisms through which fucoidans may contribute to obesity management [13].

The three extracts exhibited comparable pancreatic lipase inhibition at 1.0 mg/mL despite differences in their measured compositions and molecular weight distributions. This suggests that lipase inhibition cannot be attributed to a single measured characteristic. Further studies using purified fractions and detailed structural analysis are required to determine the respective contributions of sulphation, fucose content, molecular size and co-extracted constituents. Such observations highlight the complexity of fucoidan structure–activity relationships and support the growing consensus that multiple structural parameters collectively influence biological activity [6,24]. The results demonstrate that fucoidans from South African brown macroalgae possess appreciable pancreatic lipase inhibitory activity and further suggest that both sulphation and broader structural characteristics contribute to their anti-obesity potential.

#### 2.4.2. Fucoidan-Rich Extracts Potential in Inhibiting α-Glucosidase

**Figure 6:**
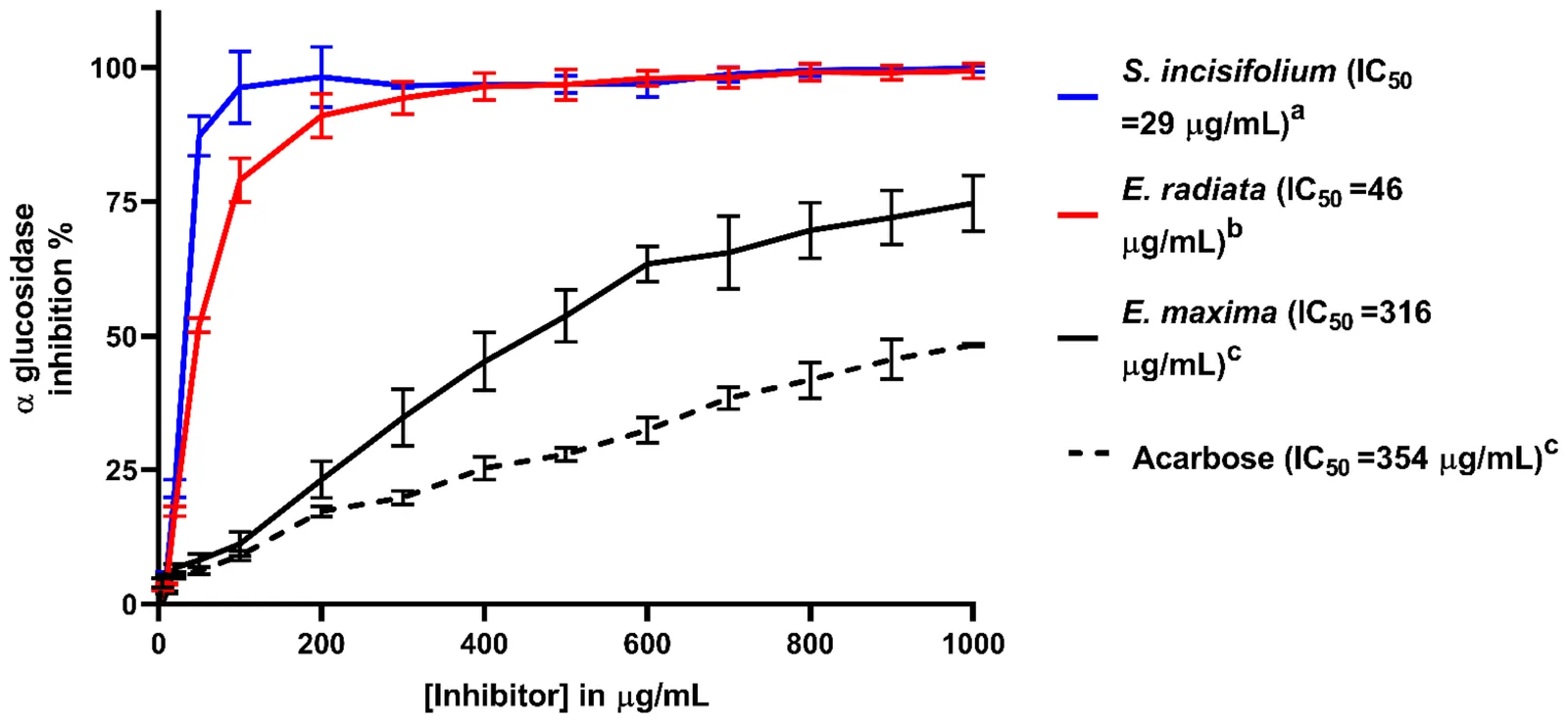
α-Glucosidase inhibitory activity of fucoidan-rich extracts from *Sargassum incisifolium*, *Ecklonia radiata*, and *Ecklonia maxima* compared with acarbose. Non-linear regression calculated IC_50_ values from GraphPad Prism v 10 are also displayed. Data are presented as mean ± SD (*n* = 3). The IC_50_ of each inhibitor was evaluated using one-way ANOVA followed by Tukey–Kramer’s multiple-comparisons test. Different lowercase letters indicate significant differences (*p* < 0.05).

The α-glucosidase inhibitory activities of fucoidans extracted from *S. incisifolium*, *E. radiata*, and *E. maxima* are presented in Figure 6. All fucoidan-rich extracts inhibited α-glucosidase activity in a dose-dependent manner; however, substantial differences in potency were observed among species. The fucoidans extracted from *S. incisifolium* and *E. radiata* exhibited exceptionally strong inhibitory activity, achieving greater than 90% inhibition at concentrations as low as 0.1 mg/mL. In contrast, *E. maxima* displayed a more gradual inhibitory response, reaching approximately 75% inhibition at 1.0 mg/mL. Acarbose, used as the positive control, exhibited considerably lower inhibitory activity throughout the tested concentration range.

The fucoidan-rich extracts investigated in the present study demonstrated potent α-glucosidase inhibitory activity, particularly those obtained from *S. incisifolium* and *E. radiata*. The IC_50_ values of 29 and 46 μg/mL, respectively, indicate markedly greater potency than acarbose (354 μg/mL) under the experimental conditions employed (Figure 6). Similar α-glucosidase inhibitory activities have been reported for fucoidans isolated from brown macroalgae, supporting the growing recognition of these sulphated polysaccharides as promising natural modulators of carbohydrate digestion [41]. However, the observed α-glucosidase inhibition cannot be attributed exclusively to the fucoidan-rich polysaccharide fraction. The extracts contained measurable polyphenols (1.08–6.76% in the dried extracts), which may also contribute to α-glucosidase inhibition. The comparatively high polyphenol content of *S. incisifolium* may therefore have contributed to its apparent potency. Further testing of polyphenol-depleted and chromatographically purified polysaccharide fractions is required to assess the contributions of fucoidan and co-extracted phenolics.

The α-glucosidase inhibitory activities of the two *Ecklonia*-derived fucoidan-rich extracts were broadly consistent with previous reports, although their quantitative potencies differed. The IC_50_ value obtained for *E. radiata* (46 μg/mL) was approximately 2.4-fold higher than the ~19 μg/mL reported by Mabate et al. (2021) [15], indicating lower potency in the present extract. In contrast, the IC_50_ value for *E. maxima* (316 μg/mL) was comparable to the previously reported range of ~270–310 μg/mL [8,15]. These quantitative differences may reflect variation in algal age, collection conditions, extraction batches, extract composition, or assay procedures. Nevertheless, the studies consistently demonstrate α-glucosidase inhibition by South African *Ecklonia*-derived extracts, supporting the reproducibility of this activity while indicating that the extent of inhibition may vary among preparations.

An important observation is that the fucoidan-rich extracts exhibited potent α-glucosidase inhibition. Similar findings have previously been reported for fucoidans from *E. radiata* and *E. maxima* [8,15]. This selective inhibition may be advantageous because excessive α-amylase inhibition can lead to gastrointestinal side effects from the fermentation of undigested carbohydrates in the colon [41]. Consequently, selective inhibition of α-glucosidase has been proposed as a preferable strategy for controlling postprandial hyperglycaemia while minimising adverse gastrointestinal effects. The results demonstrate that fucoidans extracted from South African brown macroalgae are highly effective α-glucosidase inhibitors. The exceptionally low IC_50_ values obtained for *S. incisifolium* and *E. radiata*, together with their superior performance relative to acarbose, highlight their potential as natural functional ingredients or complementary therapeutic agents for the management of postprandial hyperglycaemia and type 2 diabetes mellitus.

#### 2.4.3. Antioxidant Activity

**Figure 7:**
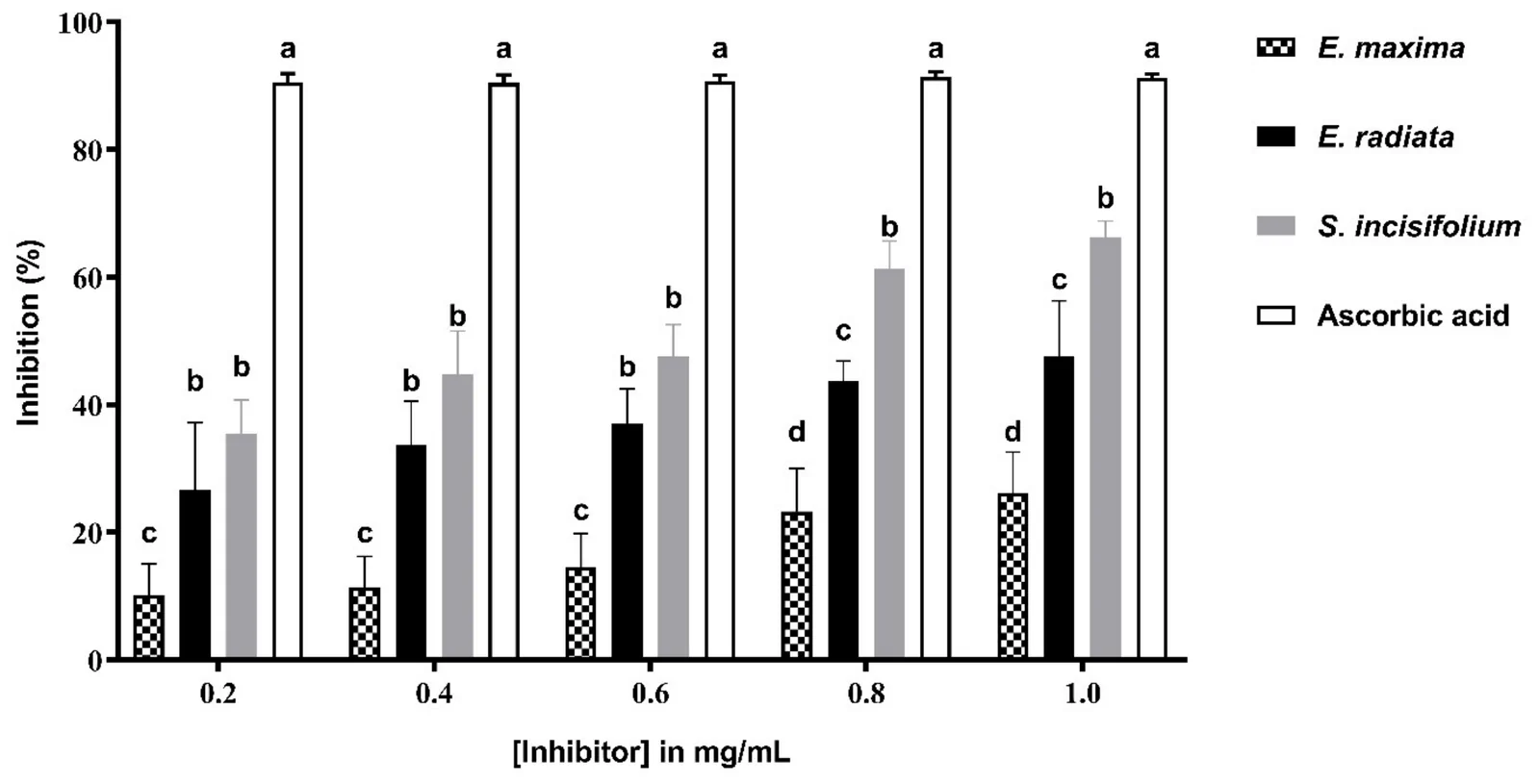
DPPH radical-scavenging activities of fucoidan-rich extracts from *Ecklonia maxima*, *Ecklonia radiata* and *Sargassum incisifolium*, compared with ascorbic acid. Values are presented as mean ± SD (*n* = 3). Differences among treatments at each concentration were evaluated using two-way ANOVA followed by Tukey–Kramer’s multiple-comparisons test. Different lowercase letters within the same concentration indicate significant differences (*p* < 0.05), whereas values sharing a letter are not significantly different.

The antioxidant activities of fucoidans extracted from *E. maxima*, *E. radiata*, and *S. incisifolium* were evaluated using the DPPH radical scavenging assay (Figure 7). All extracts exhibited concentration-dependent antioxidant activity, with radical scavenging increasing as fucoidan concentration increased. Among the investigated samples, *S. incisifolium* demonstrated the strongest antioxidant activity, achieving 66.2% DPPH radical scavenging at 1.0 mg/mL. This activity was substantially higher than that observed for *E. radiata* and *E. maxima*, which exhibited approximately 50% and 26.2% scavenging activity at the same concentration, respectively. Although the antioxidant activities of all fucoidan-rich extracts were lower than that of the positive control, ascorbic acid (91.2% inhibition at 1.0 mg/mL), the results demonstrate appreciable free-radical scavenging potential across all three species.

Among the investigated extracts, *S. incisifolium* exhibited the strongest antioxidant activity and contained the highest sulphate (~24%) and polyphenol (~6.76%) contents. Consequently, the DPPH radical-scavenging activity should be interpreted as the activity of the complete fucoidan-rich extracts and cannot be attributed specifically to the sulphated polysaccharide component. Both sulphated polysaccharides and phenolic compounds have been associated with free-radical scavenging activity in brown seaweeds [28,42]. The progressive increase in antioxidant activity from *E. maxima* to *E. radiata* and *S. incisifolium* mirrored the corresponding increases in sulphate and polyphenol content, suggesting that these constituents may collectively contribute to the observed antioxidant effects. Such complementary activity may be advantageous for nutraceutical applications, although further purification studies would be required to determine the relative contribution of each component.

#### 2.4.4. Associations Between Extract Characteristics and Bioactivity

The biological activities of fucoidans are widely recognised to be strongly influenced by their structural and physicochemical characteristics, including sulphation degree, monosaccharide composition, molecular weight, molecular weight distribution, and polysaccharide conformation [24,28]. We also deduce from our data that the presence of polyphenols may increase the bioactivity of fucoidan-rich extracts. Consequently, the characterisation performed in the present study provides valuable insight into the factors that may contribute to the observed differences in pancreatic lipase inhibition, α-glucosidase inhibition, and antioxidant activity among the investigated fucoidans.

Although all three extracts were identified as fucoidan-rich sulphated polysaccharides, notable compositional and structural differences were observed between the *S. incisifolium* and *Ecklonia* species. The fucoidan-rich extract from *S. incisifolium* was characterised by the highest sulphate content (~24.01%), the highest polyphenol content (~6.76%), the strongest sulphate-associated FTIR absorption bands, and a broader molecular weight distribution indicative of greater polydispersity. In contrast, the fucoidans extracted from *E. maxima* and *E. radiata* exhibited higher total carbohydrate and L-fucose contents, lower sulphate contents, and comparatively homogeneous molecular weight distributions. The data are consistent with previous reports demonstrating substantial interspecific variation in fucoidan composition and structure among brown macroalgae [24,29].

The antioxidant activity observed in the present study appeared to be most closely associated with sulphate and polyphenol content. *S. incisifolium* demonstrated the highest DPPH radical scavenging activity, followed by *E. radiata* and *E. maxima*, mirroring the trend observed for both sulphate and polyphenol concentrations. Sulphate groups are believed to contribute to antioxidant activity through their ability to donate electrons and stabilise reactive oxygen species, while co-extracted polyphenolic compounds may further enhance radical scavenging capacity [28,42]. Similar relationships between sulphation degree, phenolic content, and antioxidant activity have been reported for fucoidans isolated from several *Sargassum* species and other brown seaweeds [42,43].

In contrast, the relationship between fucoidan composition and metabolic enzyme inhibition appeared more complex. While *S. incisifolium* exhibited the strongest α-glucosidase inhibitory activity and contained the highest sulphate content, the *Ecklonia* fucoidans also displayed substantial enzyme inhibition despite considerably lower sulphate levels. These observations suggest that enzyme inhibition cannot be explained solely by sulphation degree and may instead arise from the combined influence of multiple structural features. Previous studies have similarly reported that fucoidan bioactivity is governed by complex interactions between sulphation patterns, monosaccharide composition, molecular weight, branching, and molecular architecture rather than any single structural parameter [6,24].

The molecular weight analysis provided further evidence of structural differences among the extracts. The Ecklonia-derived fucoidan-rich extracts had relatively lower molecular weights, while *S. incisifolium* exhibited a markedly broader molecular weight distribution than the *Ecklonia* species (Figure 1; Table 1). This greater polydispersity was reflected in the presence of both higher- and lower-molecular-weight fractions within the extract, suggesting a more heterogeneous polysaccharide population. Similar observations have been reported for fucoidans isolated from *Sargassum* species, which frequently exhibit greater structural complexity than those derived from kelps [23]. Molecular weight heterogeneity may influence polysaccharide conformation, solution behaviour, and interactions with biological targets, thereby contributing to variations in biological activity [23,26]. The NMR and FTIR analyses further supported the presence of structural differences between genera. While the fucoidans extracted from *E. maxima* and *E. radiata* exhibited highly similar spectral profiles, the *S. incisifolium* fucoidan-rich extract displayed stronger sulphate-associated signals and broader resonances within the carbohydrate region. In addition to the higher thermal stability observed during thermogravimetric analysis, these data indicate that the *S. incisifolium* fucoidan-rich extract possesses a distinct molecular organisation relative to the fucoidans isolated from the *Ecklonia* species.

The results suggest that extract bioactivity is associated with several compositional and physicochemical attributes rather than a single characteristic. The *S. incisifolium* extract showed higher sulphate and polyphenol contents, greater molecular-weight heterogeneity, and stronger antioxidant and α-glucosidase inhibitory activities. These data provide a basis for future studies using purified fractions and detailed structural analyses to determine the individual contributions of these attributes. These findings reinforce the importance of integrated structural characterisation when evaluating fucoidan functionality and contribute to the growing understanding of fucoidan structure–activity relationships in South African brown macroalgae.

## 3. Materials and Methods

### 3.1. Materials

Orlistat (CAS No. 96829-58-2), acarbose (Cat. No. A8980), α-glucosidase from *Saccharomyces cerevisiae* (Type I; Cat. No. G5003), and pancreatic lipase from porcine pancreas (Type II; CAS No. 9001-62-1) were purchased from Sigma-Aldrich (St. Louis, MO, USA). The assay substrates p-nitrophenyl-α-D-glucopyranoside (*p*NPG) and 4-nitrophenyl butyrate (*p*NPB), 2,2-diphenyl-1-picrylhydrazyl (DPPH), ascorbic acid, Triton X-100, D-glucose, L-fucose, bovine serum albumin, gallic acid, sodium sulphate, Folin–Ciocalteu reagent and Bradford reagent were also obtained from Sigma-Aldrich (St. Louis, MO, USA). Pullulan molecular-weight standards and the Shodex OHpak SB-806M HQ size-exclusion column were supplied by Showa Denko K.K. (Tokyo, Japan). Nylon membrane filters (0.22 µm) were obtained from Membrane Solutions LLC (Auburn, WA, USA). Other analytical-grade chemicals and solvents were purchased from Sigma-Aldrich (St. Louis, MO, USA), Merck KGaA (Darmstadt, Germany), as specified where applicable.

### 3.2. Seaweed Harvesting and Preparation

The fucoidan-rich extracts used in this study was extracted from three different brown macroalgae species: *E. maxima*, *E. radiata*, and *S. incisifolium*. The raw *E. maxima* kelp was kindly donated by Afrikelp (Cape Town, South Africa), while the *E. radiata* and *S. incisifolium* seaweeds were collected as beach cast from the South African Indian Ocean coastline (coordinates: 32°50′13.3″ S 28°06′58.9″ E). The macroalgae were stored on ice until arrival at the laboratory and then washed with distilled water. The samples were then cut into small pieces (approximately 5–10 cm) and dried at 40 °C in an incubator (Biobase, Jinan, China) for ~48 h with no shaking. Once dried, the seaweed was ground into a powder using a coffee grinder and stored in an airtight container at room temperature until further use. Although particle-size distribution was not measured using a defined sieve mesh, all three dried algal biomasses were milled under the same conditions to produce a visually uniform fine powder. Nevertheless, the possible influence of particle size on absolute extraction yield cannot be excluded.

### 3.3. Fucoidan Extraction

Each macroalgae sample was depigmented with absolute ethanol at a 1:20 (*w*/*v*) ratio (ground seaweed:ethanol) and incubated overnight at room temperature. Fucoidan was extracted using the hot water extraction method as described by Mabate and colleagues [15]. Briefly, the depigmented samples were combined with distilled water in a 1:30 (*w*/*v*) ratio and incubated at 70 °C overnight, with agitation. Following hot-water extraction, the suspension was centrifuged at 10,000× *g* for 10 min, and the recovered supernatant was treated with CaCl_2_ at a final concentration of 2% (*w*/*v*). The mixture was incubated at 4 °C for 4 h to precipitate alginate as insoluble calcium alginate, which was subsequently removed by centrifugation at 10,000× *g* for 10 min. The yield of extracted fucoidan-rich extract was calculated as a percentage of the dry weight of the depigmented macroalgae used in the hot-water extraction method (% dry weight).

### 3.4. Chemical Characterisation of Fucoidan-Rich Extracts

The total carbohydrate content was determined according to the Dubois method [44], using D-glucose as a standard. The L-fucose content of each sample was determined using a modified method described by Dische and Shettles [45], using L-fucose as the standard. The sulphate content of each sample was determined after desulphation with 60% formic acid (*v*/*v*). A modified barium chloride-gelatine method was used for quantification, with sodium sulphate as a standard [46]. The protein content of each sample was determined using the Bradford method [47], with bovine serum albumin (BSA) as a standard. The total phenolic content of each sample was determined using a modified Folin-Coicalteu method [48]. Gallic acid was used as a standard.

### 3.5. Fucoidan-Rich Extract Molecular Weight Determination

The molecular weights of the fucoidan-rich extracts were determined by size-exclusion high-performance liquid chromatography coupled with a refractive index detector (HPLC-RID). Separation was performed on a Shodex OHpak SB-806M HQ column (8.0 mm I.D. × 300 mm; Showa Denko, Tokyo, Japan) according to the manufacturer’s recommendations. The mobile phase, consisting of 0.1 M aqueous NaNO_3_, was filtered through 0.22 µm nylon membranes (Membrane Solutions, Auburn, WA, USA) prior to use. Chromatographic analysis was carried out at a flow rate of 0.5 mL/min, with the column maintained at 30 °C and an injection volume of 20 µL. Pullulan standards (Shodex, Tokyo, Japan) were used to generate a calibration curve for the determination of fucoidan molecular weights.

### 3.6. FTIR Analysis

Fourier-transform infrared spectroscopy (FTIR) analysis was performed using a PerkinElmer Spectrum 100 FTIR spectrometer (PerkinElmer, Wellesley, MA, USA) operated in attenuated total reflection (ATR) mode. Approximately 100 mg of each fucoidan-rich extract was analysed over a wavenumber range of 4000–600 cm^−1^ with spectra acquired by averaging 16 scans. Baseline and ATR corrections for penetration depth and frequency variations were carried out using Spectrum One software (version 1.2.1) (PerkinElmer, Wellesley, MA, USA).

### 3.7. NMR Spectroscopy Analysis

Fucoidan-rich extracts (10 mg) were dissolved in 1 mL D_2_O, centrifuged at 13,000× *g* for 2 min, and filtered through a 0.45 μm membrane filter prior to analysis. ^1^H NMR spectra were recorded at 23 °C using a Bruker 400 MHz spectrometer (Bruker, Fällanden, Switzerland) equipped with TopSpin 3.5 software (Bruker, Billerica, MA, USA).

### 3.8. Thermogravimetric Analysis

Thermogravimetric analysis (TGA) was performed using a PerkinElmer TGA 4000 thermogravimetric analyser (PerkinElmer, Waltham, MA, USA) to evaluate the thermal stability and ash content of the fucoidan-rich extracts. Approximately 2–5 mg of each sample was placed in an aluminium pan and heated from 30 to 550 °C at a heating rate of 15 °C/min under an inert nitrogen atmosphere (99.99% purity) with a flow rate of 20 mL/min. A separate blank run using an empty pan was conducted for baseline correction. The resulting weight loss was expressed as a percentage relative to temperature increase, and derivative thermogravimetric (DTG) curves were generated using GraphPad Prism version 9 (GraphPad Inc., San Diego, CA, USA).

### 3.9. Inhibition of Pancreatic Lipase

The inhibitory potential of the three fucoidan extracts against pancreatic lipase was investigated by carrying out an enzyme inhibition assay using *p*-nitrophenyl butyrate (*p*NPB) as a substrate. Fucoidan-rich extracts (0.1–1.0 mg/mL), pancreatic lipase (0.5 mg/mL), and *p*NPB (1 mM) were combined in 50 mM phosphate buffer (pH 7.0) supplemented with Triton X (0.5% *v*/*v*) to make up a total reaction volume of 250 µL. Orlistat (1.0 mg/mL), a known pancreatic lipase inhibitor, was dissolved in water and DMSO (1:1) and used as a positive control. The reaction was incubated for 30 min at 37 °C, and the absorbance at 405 nm was read using a microplate reader (Hangzhou Allsheng Instruments, Hangzhou, China). The remaining activity of pancreatic lipase in per cent (%) was calculated relative to the uninhibited control.

### 3.10. Inhibition of α-Glucosidase

The inhibitory effect of the fucoidan-rich extracts on α-glucosidase was investigated by measuring α-glucosidase activity in the presence of these potential inhibitors. Fucoidan-rich extracts (0.005–1.0 mg/mL) were combined with 25 mM *p*-nitrophenyl-α-D-glucopyranoside (*p*NPG) substrate in 50 mM sodium phosphate buffer (pH 7.0). Acarbose (0.1–1.0 mg/mL), a known α-glucosidase inhibitor, was used as a positive control. The reaction was initiated by adding α-glucosidase (6.25 µg/mL) to a final reaction volume of 250 µL. The reaction was incubated for 20 min at 37 °C, and absorbance at 405 nm was measured to quantify the release of *p*-nitrophenol. The inhibition percentage (%) of α-glucosidases was calculated relative to the uninhibited control.

### 3.11. DPPH Radical Scavenging Assay

The antioxidant activity of the fucoidan-rich extracts was determined using the DPPH radical scavenging assay. Fucoidan-rich samples (0.2–1.0 mg/mL) were incubated with 0.004% (*w*/*v*) DPPH solution in methanol at room temperature for 30 min in the dark. Ascorbic acid (0.2–1.0 mg/mL) served as a positive control. Absorbance was measured at 517 nm, and antioxidant activity was determined from the decrease in DPPH radical absorbance following reaction with the fucoidan-rich extracts.

### 3.12. Statistical Analysis

All experiments were performed using at least three independent biological replicates (n ≥ 3), unless otherwise stated. Data were presented as the mean ± standard deviation (SD). Statistical analyses were performed using GraphPad Prism version 10 (GraphPad Software, Boston, MA, USA). Comparisons of individual chemical and compositional parameters among the *S. incisifolium*, *E. radiata*, and *E. maxima* extracts were conducted using one-way analysis of variance (ANOVA), followed by Tukey’s multiple-comparisons test. IC_50_ values were determined by nonlinear regression using a variable-slope concentration–response model and are reported with 95% confidence intervals. All compositional analyses were performed using three independent biological replicates (*n* = 3). Because this sample size provides limited power to reliably assess normality and homogeneity of variance, the results of the one-way ANOVA and Tukey multiple-comparisons test should be considered preliminary and interpreted with caution. Differences were considered statistically significant at *p* < 0.05.

## 4. Conclusions and Future Perspectives

Fucoidan-rich extracts were successfully obtained from the South African brown macroalgae *E. maxima*, *E. radiata*, and *S. incisifolium*. Their compositional and spectroscopic characteristics were consistent with fucose-containing sulphated polysaccharides, although clear species-dependent differences were observed in carbohydrate, L-fucose, sulphate, and polyphenol contents, molecular weight distribution, thermal behaviour, and spectral profiles. *S. incisifolium* produced a particularly distinct extract characterised by higher sulphate and polyphenol contents, greater molecular-weight heterogeneity, enhanced thermal stability and stronger DPPH radical-scavenging and α-glucosidase-inhibitory activities. All three extracts also demonstrated concentration-dependent inhibition of pancreatic lipase. The observed associations suggest that sulphation, polyphenol content and molecular-weight distribution may collectively influence the in vitro biological activities of these extracts; however, causative structure–activity relationships cannot be established from the present analyses. Furthermore, the use of fucoidan-rich rather than purified fractions, the partial structural characterisation of the extracts, and the reliance on in vitro assays represent important limitations of this study. Consequently, co-extracted compounds may have contributed to the activities, and causal structure–activity relationships cannot be established. Further purification, structural analysis, and biological validation are required. Future studies should fractionate the extracts, determine their complete monosaccharide and linkage profiles, and evaluate the biological activities of the resulting purified fractions. These findings identify South African brown macroalgae as promising sources of fucoidan-rich extracts for further investigation as anti-hyperglycaemic agents, functional food ingredients, and nutraceuticals.

## Figures and Tables

**Table 1:** Chemical characteristics of the dried fucoidan-rich extracts.

Parameter	*Sargassum* *incisifolium*	*Ecklonia* *radiata*	*Ecklonia maxima*
**Extraction yield** (g extract/100 g depigmented macroalgae)	3.73 ± 0.54 ^b^	3.92 ± 0.49 ^b^	8.76 ± 0.81 ^a^
**Total carbohydrate** (g GE/100 g dried extract)	38.50 ± 4.57 ^b^	90.23 ± 3.69 ^a^	96.27 ± 4.79 ^a^
**Total phenolic content** (g GAE/100 g dried extract)	6.76 ± 0.85 ^a^	4.02 ± 1.08 ^b^	1.08 ± 0.53 ^c^
**Protein** (g BSAE/100 g dried extract)	6.43 ± 0.43 ^a^	6.75 ± 1.69 ^a^	4.76 ± 1.74 ^a^
**Sulphate** (g sulphate equivalents/100 g dried extract)	24.01 ± 3.36 ^a^	15.27 ± 0.92 ^b^	10.70 ± 1.04 ^b^
**L-fucose** (g L-fucose equivalents/100 g dried extract)	36.74 ± 1.84 ^b^	55.86 ± 3.77 ^a^	49.55 ± 2.64 ^a^
**Total uronic acids** (g uronic acid equivalents/100 g dried extract)	1.2 ± 0.2 ^b^	2.7 ± 0.6 ^a^	2.2 ± 0.3 ^ab^
**Ash** (g/100 g dried extract)	37.6 ± 1.2 ^a^	29.4 ± 2.4 ^b^	24.5 ± 1.42 ^c^

Values are represented as means ± SD (*n* = 3 independent biological replicates) and are expressed on a dried-extract basis. Different superscript lowercase letters within the same row indicate significant differences among extracts (one-way ANOVA followed by Tukey’s multiple-comparisons test, *p* < 0.05). Values that share at least one letter are not significantly different. GE, glucose equivalents; GAE, gallic acid equivalents; BSAE, bovine serum albumin equivalents. Assay-derived values are not mutually exclusive and should not be summed as an additive mass balance.

## Data Availability

The data presented in this study are available on request from the corresponding author.
